# Microbial analysis and virulence genes detection of milk preserved using heat-assisted pulsed electric field

**DOI:** 10.1186/s13104-021-05805-3

**Published:** 2021-10-26

**Authors:** Suci Yuliangsih, Diana Elizabeth Waturangi, Yogiara

**Affiliations:** 1 Indonesian Food and Drug Authority, Jalan Percetakan Negara No. 23, Jakarta Pusat, 10560 Indonesia; 2 Faculty of Biotechnology, Atma Jaya Catholic University of Indonesia, Jalan Jenderal Sudirman 51, Jakarta, 12930 Indonesia

**Keywords:** Microbial analysis, Virulence genes, PEF, Milk, Heating

## Abstract

**Objective:**

Microbial analysis in milk preserved using heat-assisted Pulsed Electric Field (PEF) need to be assessed. In this study we analyze the microbial quality and virulence-associated genes in milk samples preserved using heat-assisted PEF from several producers in Indonesia.

**Results:**

Milk samples were collected consisting of raw milk, milks taken after the heating, PEF, mixing, cooling, and packaging. Microbiological and Polymerase Chain Reaction (PCR) detection for virulence genes were performed. Heat-assisted PEF treatment gave 2.7–7.47 log reduction for TPC; 1.6–2.56 log reduction for MPN number; 3.13–6.48 log reduction for *S. aureus*; and for *B. cereus* there was an increase of 0.76 log and a reduction of 0.46 log. While milk samples from thermal pasteurization gave log reduction numbers of TPC, MPN, and *S. aureus* respectively 5.28; 2.56; and 4.73, for *B. cereus* was increasing 2.4 log. Producer C performed the best results with significant reduction compared with others (p < 0.005). There were no colonies of *L. monocytogenes* found in all of the samples. PCR results showed that milk samples possessed virulence genes 17.5% (10/57) of *invA* genes, 54.4% (31/57) of *nheA* genes, 68.4% (39/57) of *cytK* genes, 38.6% (22/57) of *nuc* genes, 63.2% (36/57) of *ileS* genes, while *hly* and *actA* genes were not detected.

**Supplementary Information:**

The online version contains supplementary material available at 10.1186/s13104-021-05805-3.

## Introduction

Globally, billions of people are at risk of foodborne diseases (FBDs) [1]. Milk and dairy products can harbor a variety of microorganisms and can be important sources of FBDs [2]. Microbes that may be present in milk can include pathogens, spoilage organisms, organisms that may be conditionally beneficial [3]. Microbes that contaminate milk have some virulence genes such as: *Salmonella* spp. with invasion A (*invA*) gene that responsible for initiating infection [4]; *B. cereus* can produce enterotoxin, Non Hemolytic Enterotoxin (NHE) and Cytotoxin K encoded by *nheA cytK* [5]; *L. monocytogenes* contains listeriolysin which lyses erythrocytes and other cells, it encoded by *hly,* while *actA* gene to promote intracellular motility [6]. For the *nuc* gene encoded staphylococcal thermonuclease is a biofilm inhibitor that degrades environmental DNA (eDNA) associated with biofilm; [7], while some *Staphylococcus* resistance to mupirocin which inhibits protein synthesis by binding to the bacterial isoleucyl-tRNA synthetase enzyme which is encoded by the *ileS* gene [8].

Non-thermal preservation methods have a minimal impact on the sensory, quality and nutritional status of food. Pulsed Electric Field (PEF) provides an alternative choice for various food products particularly for liquid foods, which provide better preservation and maintenance of fresh-like quality aspects of food [9].

During milk preservation, contamination could be happened from bulk tank milk occurs during and after milking; thermoduric bacteria in milk that can arise from the soil, bedding, feed, dust, all of which contaminate cow's teats, and also occur from deposits on milking equipment [10].

PEF treatment is defined as applying a short burst of high voltage electric pulses in the range of 20–80 kV cm^−1^ placed between two electrodes is involved in pulsed electric fields processing. Treatment time of PEF lasts in microseconds; thus, the increase in treatment temperature during PEF processing is minimized. The application of high voltage results in an electric-fields that causes microbial inactivation [11]. Some PEF instruments have been developed in Indonesia and the microbial quality had been assessed and showed that PEF could reduce total microbe from 7.8 × 10^5^ CFU/ml to 3.09 × 10^2^ CFU/ml (49.48 kV; 270 s) [12]. While Muslim et al.[13] stated that PEF can reduce the number of *S.aureus* in fresh milk from 1.6 × 10^3^ CFU/ml to 1.157 × 10^3^–3.97 × 10^2^ CFU/ml. However, microbial analysis for other bacteria as well as determination of their virulence potential, which are commonly associated with milk contamination is very limited. Milk preserved using PEF in Indonesia are consumed daily by some children. Pathogenic microbial contamination of milk may lead to infection in pediatric population [14]. Indonesian Food and Drug Authority [15] has determined the maximum limit of microbiological contamination for pasteurized milk, which include: total plate count (TPC), Enterobacteriaceae and *Salmonella* respectively m = 10^4^, M = 10^5^ colony/ml; m =  < 1 MPN/ml, M = 5 MPN/ml; and negative/25 ml. This research aimed to analyze the microbiological hazards at small-medium enterprises from raw milks to packaged milks after that detected the virulence-associated genes of the isolates.

Microbiological hazards analysis of PEF milk is still limited in Indonesia. Thus, this study would be useful for FBDs prevention, support microbiological risk assessment, policy-making, and improvement the process at SMEs.

## Main text

### Methods

#### Bacterial strains and culture medium

Bacterial strains used in this study were *Salmonella* Typhimurium (ATCC 14028), *Listeria monocytogenes* (ATCC 7644)*, Staphylococcus aureus* (ATCC 25923)*, Bacillus cereus* (ATCC 11778), *Bacillus subtilis* (ATCC 6633 NCTC 10400)*,* and *Escherichia coli* (25922) (Indonesian FDA Culture Collection). All of the strains were grown in Tryptone Soya Broth at 37 °C for 24 h. Grown cultures were streaked on selective medium agar respectively, Xylose Lysine Desoxycholate Agar, PALCAM Agar, Baird Parker Agar, Mannitol Egg Yolk Polymixin Agar, and Eosin Methylene Blue Agar to verify the purity. Single colonies were picked from agar plates and streaked on Tryptone Soya Agar then incubate over night at 37 °C.

#### Sampling

A total 57 milk samples were taken from four small medium enterprises at A, B, C, and D located in Jakarta and East Java, Indonesia, for each process including: raw milk, heated milk, PEF milk, cooled milk, and packaged milk. Samples were poured into sterile tubes then chilled and brought immediately to the laboratory for further assays. UHT milk was also taken from the supermarket to be tested in the laboratory as a comparison.

#### Microbiological analysis

Microbiological analyses were performed to confirm the presence of pathogenic bacteria. 25 ml of samples were suspended in 225 ml of bacterial medium to obtain a 1:10 dilution and treated based on SNI 2897:2008 [16] and ISO 7218:2007 [17] for testing TPC, *S. aureus, L. monocytogenes, Salmonella* spp; ISO 21528–1:2004 [18] for testing MPN Enterobacteriaceae and SNI ISO 7932:2012 [19] for testing *B. cereus.* Data were analyzed using variance analysis if the ANOVA test showed statistically significant difference, then Tukey test was further carried out to determine whether there was a difference between each treatment.

#### Molecular analysis

DNA extraction was done using ZymoBIOMICS™ DNA Mini Kit method for milk samples and the boiling method for bacteria isolates. Five specific colonies were selected randomly using sterile loop and streaked on BHI agar, then incubate at 37 °C for 24 h. Colonies were picked and put into 500 µl of sterile NaCl. Samples were boiled for 15 min, then centrifuged at 12.000 rpm for 5 min. The supernatant was taken and transferred into a new tube. DNA concentration was measured using BioDrop Duo UV/Vis spectrophotometer, and then the supernatant was used as a DNA template for PCR detection [20]. A preliminary study was done to optimize PCR condition and specificity test. PCR amplification was performed using Go Taq Green Master Mix (Promega, USA). PCR reactions and protocol were informed in Additional file 1: Table S1.

### Results

#### Microbiological analysis

Results showed that combination of heating (50–74 °C for approximately 5 s) and PEF process could reduce log cycles number of TPC, MPN, *S. aureus* and *B. cereus* respectively at producer A (7.47, 2.56, 4.67, 0.46); producer B (2.86, 1.6, 6.48, − 0.76); producer C (2.7, 1.68, 3.13, 0); and producer D (thermal pasteurization) (5.28, 2.56, 4.73, 0); while in negative control (sample E) there were no microorganisms growing in the media.

#### Statistical analysis

Statistical analysis was done by using quantitative microbiological results from final products. The results showed that independent variables (pasteurization process) have a statistically significant effect on the dependent variables in mean interest (*p* < 0.001) between among dependent variables. Then it was continued with LSD and Tukey analysis. Multiple comparisons showed that the error term was 0.737 and the mean difference was significant at the 0.05 level. It was described in more detail in subset on Additional file 2: Table S2.

Based on Additional file 2: Table S2, milk processing established at producer C showed the best results compare with other producers based on significance result and also showed the lowest total number of microorganism found from TPC assay for *S. aureus* and *B. cereus* compared with other producers.

#### Molecular analysis

Some virulence genes were detected in processed samples and also final products of producers A, B, C and D (Fig. 2). PCR results revealed that milk samples possessed virulence genes 17.5% (10/57) *invA* genes in raw milk and packaged flavor milk; 54.4% (31/57) *nheA* genes in milk samples after heating, PEF, cooling and packaging process; 68.4% (39/57) *cytK* genes in all milk samples; 38.6% (22/57) *nuc* genes in raw milk, after heating and packaging process; 63.2% (36/57) *ileS* genes in raw milk, after heating, PEF and packaging process; while *hly* and *actA* genes were not detected in this study. Packaged milk with flavour gave the highest detection of *invA, nheA,* and *cytK*. This step could be identified as a contamination source in milking production using PEF techniques.

### Discussion

#### Microbiological analysis

**Fig. 1:**
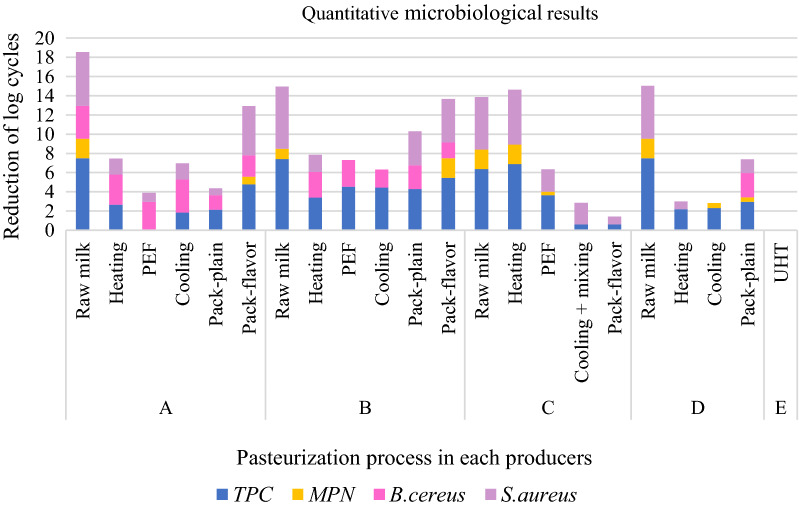
Quantitative microbiological results. * Raw milk were included in this picture to give comparison of reduction after the treatment

Based on our result combination of heating and PEF could reduce number of bacteria (Fig. 1). In the framework of the hurdle concept, several researches have been focused on the use of PEF in combination with heat and/or antimicrobials to increase its efficacy [21]. In another trial, PEF processing of milk was combined with heat treatment up to 55–60 °C and a significant reduction was observed in the microbial load [9]. The experiment of Hawa et al.[22] showed that the highest *E. coli* inactivation was a combination of thermal (70 °C, 15 s) and PEF (100 kV, 24.37 s) can reduce *E. coli* 2.5 log cycles in fresh milk.

*Salmonella* can enter milk from raw or improperly pasteurized milk and eggs, as well as other food have also been associated with salmonellosis or causing diseases [23, 24]. Kamana et al. [25] stated that 21.4% of the boiled milk sold from milk shops was *Salmonella* positive, clearly indicating as Post Pasteurization Contamination (PPC). Several Gram-negative bacteria, when introduce as PPC, can grow rapidly at refrigeration temperatures around 6 °C and can increase bacterial contamination above 20,000 CFU/ml and spoilage that can be detected sensorily within 7 to 10 d of processing [26].

Figure 1 showed the number of *S. aureus* increased after packaging step. Contamination could happen during the addition of flavor and packaging steps. *B. cereus* have the ability to form spores when subjected to harsh environment, extreme pH values or nutrition lacking environment. *Listeria* is one of the most resistant vegetative cells populations to a PEF treatment at neutral pH while *S. aureus* can produce a toxin in improperly stored food that, if ingested, will produce mild to severe symptoms [10, 27–29].

PEF protocols from each producers showed that not necessarily effective in destroying all kinds of milk microbes. It has been observed that there is a great variation in the sensitivity of different strains of the same species of bacteria to PEF treatment [23]. Furthermore, Gram-positive bacteria showed more resistance to PEF than Gram-negative [24], *Salmonella* and *E. coli* are more susceptible to PEF as compared to *Listeria* and *Bacillus* species. The presence of fat and protein moieties limits the adeptness of PEF in whole milk because these molecules protect bacterial cells during treatment [9]. The microbiological quality of dairy products reflects good hygienic practices during the dairy milking process [30].

#### Molecular analysis

*Salmonella* spp. gene *invA* was detected in colonies of raw milk and some final products at producer B (Table 1). Jasim et al. [31] found 100% virulence genes include *invA* gene in milk samples: raw milk (cow), raw milk (street vendors and shops); and processed milk (pasteurized milk); Omar et al. [32] detected both *invA* and *stn* genes in all representative *Salmonella* serovar isolated from milk and dairy products; and Liwan et al. [30] stated that virulence factor *invA* were detected in all raw milk samples.

**Table 1:** Effects of PEF process to reduce microbe contamination in milk

Producers	Process	TPC	MPN	*Salmonella* spp.	*S. aureus*	*B. cereus*	*L. monocytogenes* (qualitative)
		(colony/ml)	Enterobacter	(qualitative)			
			(MPN/ml)				
A	Raw milk	> 3 × 10^7^	> 110	V	4 × 10^5^	2.7 × 10^3^	Less than 1
	Heating (60 °C)	4.7 × 10^2^	< 0.3	Less than 1	4.8 × 10^1^	1.3 × 10^3^	Less than 1
	PEF	less than 1	< 0.3	Less than 1	8.5	9.3 × 10^2^	Less than 1
	Cooling (50 oC)	7.1 × 10^1^	< 0.3	Less than 1	5.1 × 10^1^	2.6 × 10^3^	Less than 1
B	Raw milk	2.5 × 10^7^	12	V	> 3 × 10^6^	< 100	Less than 1
	Heating (74 °C)	2.6 × 10^3^	< 0.3	Less than 1	6.3 × 10^1^	4.5 × 102	Less than 1
	PEF(50 kV, 10 s)	3.5 × 10^4^	< 0.3	Less than 1	less than ^1^	5.8 × 102	Less than 1
	Cooling (50 °C )	2.8 × 10^4^	< 0.3	Less than 1	less than ^1^	7.2 × 101	Less than 1
C	Raw milk	2.3 × 10^6^	> 110	Less than 1	2.8 × 10^3^	less than 1	Less than 1
	Heating (60 °C)	7.8 × 10^6^	> 110	Less than 1	4.9 × 10^5^	less than 1	Less than 1
	PEF 1	3.5 × 10^5^	> 110	Less than 1	2.5 × 10^5^	less than 1	Less than 1
	PEF 2 (55 °C)	1.5 × 10^5^	> 110	Less than 1	2.4 × 10^3^	less than 1	Less than 1
	PEF 3 (60 °C)	4.6 × 10^3^	2.3	Less than 1	2.1 × 10^2^	Less than 1	Less than 1
	Mixing (50 °C)	< 4	< 0.3	Less than 1	1.8 × 10^2^	Less than 1	less than 1
D	Raw milk	> 3 × 10^7^	> 110	V	3.4 × 10^5^	Less than 1	Less than 1
	Thermal Pasteurization (65–70 °C)	1.6 × 10^2^	< 0.3	Less than 1	6.3 × 10^0^	Less than 1	Less than 1
	Cooling (room temperature)	2 × 10^2^	3.5	Less than 1	Less than 1	Less than 1	Less than 1
E	UHT milk	Less than 1	Less than 1	Less than 1	less than 1	Less than 1	Less than 1
	UHT milk	Less than 1	Less than 1	Less than 1	less than 1	Less than 1	Less than 1

*Bacillus cereus* produces three secreted pore-forming cytotoxin Hbl, Nhe and cytK have been implicated as the causative agents of diarrheal disease [33, 34]. The non-hemolytic enterotoxin (Nhe) is responsible for diarrhea [35]. Both *nheA* and *cytK* were detected in samples from producer A, B, and D, while it was not found at producer C (Table 1). Saleh-Lakha et al. [36] reported that more than 5.5% of moderately temperature-abused products (store at 7 °C) were found to contain > 10^5^ CFU/ml *B. cereus.* Amor et al. [5] reported that *nheA* (98.9%) and *cytK* (37.9%) were detected among *B. cereus* isolated from Tunisian foodstuffs.

**Fig. 2:**
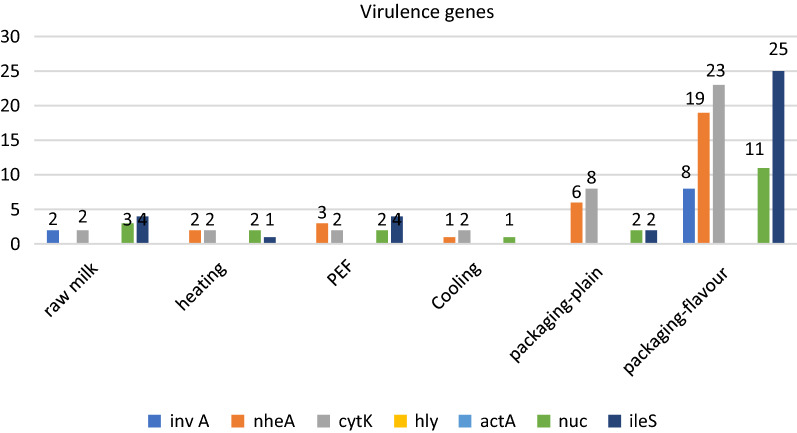
Virulence genes detection. invA for Salmonella; nheA and cytK for B. cereus; hly and actA for *L. monocytogenes*; nuc and ileS for *S. aureus*

*Staphylococcus aureus* specific gene *nuc* and *ileS* were detected in some milk samples as well as PCR detection from colonies (Fig. 2). *S. aureus* usually contaminate dairy products because it has a wide range living environment and can cause mastitis diseases which is still a problem in some dairy farms. Although *L. monoctogenes* specific gene *hly* and *actA* were not detected in all of samples, it can grow in refrigerated temperatures, which makes this organism a particular problem for food industry [37]. PEF producers must apply GMPs and follow recommendation guidelines of NACMF as an effort to create quality and safe products.

### Conclusion

PEF has the potential to reduce microbial contamination in milk, but some microorganisms were resistant to PEF treatment. Combination of PEF with heating increase the efficacy. Monitoring of the quality need to be performed included post PEF treatment.

## Limitations

Number of samples need to be increase. Effect of PEF on protein content have not been elaborated. PCR products need to be confirmed with DNA sequencing.

## Supplementary Information

**Additional file 1: Table S1.** Primer sequences and PCR reactions.

**Additional file 2: Table S2.** Subset of significant difference in final products on each process.

## Data Availability

The datasets used and/or analysed during the current study are available from the corresponding author on reasonable request.

## Abbreviations

SNI: Indonesian National Standard
DNA: Deoxyribonucleic acid
GMPs: Good Manufacturing Practices
NACMF: National Advisory Committee on Microbiological Criteria for Foods
