# Supplementary material for: Microbial analysis and virulence genes detection of milk preserved using heat-assisted pulsed electric field
Source: BMC Res Notes. 2021 Oct 26;14:397. doi: 10.1186/s13104-021-05805-3 (PMC8549208; doi:10.1186/s13104-021-05805-3)
Supplement: Supplementary file 1 — Additional file 1: Table S1. Primer sequences and PCR reactions. [file 13104_2021_5805_MOESM1_ESM.docx]

Table S1 Primer sequences and PCR reactions

| **Microorganisms** | **Primer sequence (5′- 3′)** | **Primer**  **conc.** | | **PCR Protocols** | | **Target gene** | | **Product size (bp)** | | **Reff.** | |
| --- | --- | --- | --- | --- | --- | --- | --- | --- | --- | --- | --- |
| *Salmonella* spp | CATACAGCACCAGGTCACGGGGAA  GTTCTCCAGTCGTGTGGATAGC | 0,2 μM  0,2 μM | *1 | | *invA* | | 284 | | [21] | |  |
| *L. monocytogenes*  (duplex PCR) | TAGTTCGCTGAATAGTGGCGA  TTGCTTTTTCGTCTTCTGCAC | 0.4 μM  0.4 μM | 2 | | *actA* | | 611 | | | [22] | |
|  | TGCAAGTCCTAAGACGCCAA  CCACACTTGAGATATATGCAGGA | 0.4 μM  0.4 μM |  |  | *hly* | | 753 | | | [23] | |
| *B.cereus*  (duplex PCR) | GGCGCTAGTGCAACATTACG  TCATACCAGGAGAGAAACCGC | 0.3 μM  0.3 μM | 3 | | *cytK* | | 482 | | | [23] | |
|  | AAGGCGAATGTACGAGAGTGG  CTTCTCTCGTTTGACTATCTGCAG | 0.4 μM  0.4 μM |  |  | *nheA* | | 553 | | | [23] | |
| *S. aureus*  (singleplax PCR) | CATACAGCACCAGGTCACGGGGAA  GTTCTCCAGTCGTGTGGATAGC  GCGATTGATGGTGATACGGTT  AGCCAAGCCTTGACGAACTAAAGC | 0.3 μM  0.3 μM  0.4 μM  0.4 μM | 4  5 | | *ileS*  *nuc* | | 227  280 | | | [22]  [24] | |

*1:Initial denaturation 95 ^o^C for 5 min, 35 cycles of denaturation 95 ^o^C for 1 min, annealing 60 ^o^C for 1 min,extension 72 ^o^C for 1 min, and final extension 72 ^o^C for 4 min; 2: Initial denaturation 94 ^o^C for 5 min, 30 cycles of denaturation 94 ^o^C for 1 min, annealing 58 ^o^C for 1 min, extension 72 ^o^C for 1.30 min, and final extension 72 ^o^C for 6 min; 3: Initial denaturation 94 ^o^C for 5 min, 30 cycles of denaturation 94 ^o^C for 1 min, annealing 56 ^o^C for 1 min, extension 72 ^o^C for 1.30 min, final extension 72 ^o^C for 6 min; 4: Initial denaturation 94 ^o^C for 5 min, 40 cycles of denaturation 94 ^o^C for 45 s, annealing 56 ^o^C for 1 min, extension 72 ^o^C for 45 s and final extension 72 ^o^C for 10 min; 5: Initial denaturation 94 ^o^C for 5 min, 30 cycles of denaturation 94 ^o^C for 1 min, annealing 55,1 ^o^C for 1 min, extension 72 ^o^C for 2 min, and final extension 72 ^o^C for 10 min
