# Supplementary material for: Microbial analysis and virulence genes detection of milk preserved using heat-assisted pulsed electric field
Source: BMC Res Notes. 2021 Oct 26;14:397. doi: 10.1186/s13104-021-05805-3 (PMC8549208; doi:10.1186/s13104-021-05805-3)
Supplement: Supplementary file 2 — Additional file 2: Table S2. Subset of significant difference in final products on each process. [file 13104_2021_5805_MOESM2_ESM.docx]

Table S2 Subset of significant difference in final products on each process

| TPC Process | N | Subset | | |
| --- | --- | --- | --- | --- |
|  |  | 1 | 2 | 3 |
| C | 5 | 0.6000 |  |  |
| D | 2 |  | 2.9500 |  |
| A | 12 |  | 3.6758 |  |
| B | 16 |  |  | 5.4756 |
| Sig. |  | 1.000 | 0.539 | 1.000 |
| MPN Process | N |  | |  |
|  |  | 1 | 2 |  |
| D | 2 | 0.4750 |  |  |
| C | 5 | 0.5600 |  |  |
| A | 12 | 0.5867 |  |  |
| B | 16 |  | 2.5050 |  |
| Sig. |  | 0.958 | 1.000 |  |
| *S.aureus* Process | N |  | |  |
|  |  | 1 | 2 |  |
| C | 5 | 0.8140 |  |  |
| D | 2 | 1.4400 |  |  |
| A | 12 | 3.2875 | 3.2875 |  |
| B | 16 |  | 4.5300 |  |
| Sig. |  | 0.051 | 0.534 |  |
| *B.cereus* Process | N |  | |  |
|  |  | 1 | 2 |  |
| C | 5 | 0.0000 |  |  |
| B | 16 | 1.3856 | 1.3856 |  |
| A | 12 |  | 1.9317 |  |
| D | 2 |  | 2.5100 |  |
| Sig. |  | 0.083 | 0.205 |  |
